# On the Treatment of Pulmonary Consumption

**Published:** 1872-03

**Authors:** 


*On the treatment of Pulmonary Consumption by Hygiene, Climate and Medicine, in its connexion with modern doctrines.* By James Henry Bennet, M. D. Second edition. New York: D. Appleton & Co., 1872.

This is a very interesting and valuable monograph, which places the ideas of the author in a clear and plain manner before the profession. The views of the author on the origin of Pulmonary Consumption, are those generally adopted by the profession, namely, those of Prof. Bennett of Edinburgh. His treatment of Consumption embraces those great requisites to life and health; cleanliness, fresh air, good food, and out door life.

We can not but think that if these ideas were more fully carried out, more success would attend the treatment of this disease. We recommend the work to the careful consideration of the profession.

